# *Brugia malayi* infection in ferrets – A small mammal model of lymphatic filariasis

**DOI:** 10.1371/journal.pntd.0006334

**Published:** 2018-03-30

**Authors:** Belinda M. Jackson-Thompson, So Young Kim, Shalini Jaiswal, Jessica R. Scott, Scott R. Jones, C. Paul Morris, J. Judd Fite, Karen Laurie, Andrew R. Hoy, Bernard J. Dardzinski, Edward Mitre

**Affiliations:** 1 Department of Microbiology and Immunology, Uniformed Services University, Bethesda, Maryland, United States of America; 2 Department of Radiology and Radiological Science, Uniformed Services University, Bethesda, Maryland, United States of America; 3 Navy Medicine East (M3), Portsmouth, Virginia, United States of America; 4 Department of Pathology, Johns Hopkins University School of Medicine, Baltimore, Maryland, United Sates of America; 5 WHO Collaborating Centre for Reference and Research on Influenza at the Peter Doherty Institute for Infection and Immunity, Melbourne, Australia; University of Liverpool, UNITED KINGDOM

## Abstract

**Background:**

The lack of effective short-course therapies for treatment of the adult stage of filarial worms is a major limitation in the global effort to eliminate lymphatic filariasis. Studies using current small mammal models of lymphatic filariasis are limited by difficulties in quantifying adult worm numbers and in assessing lymphatic anatomy and function.

**Methodology/Principal findings:**

Here, we re-established *Brugia malayi* infection of ferrets as a model for lymphatic filariasis and demonstrated parasitological, immunological, and histological parallels with human infection. Subcutaneous injection of L3 larvae into a hind-footpad resulted in a mean of 18 adult worms recovered 16 weeks post-infection, primarily from the draining inguinal and femoral lymphatics of the injected limb. Infected ferrets developed microfilaremia, with patency lasting from 12–26 weeks post-infection. Quantitative PCR assessing cytokine transcription by antigen-stimulated lymph node cells demonstrated a mixed Th1/Th2 response occurring during early infection. Immunoregulation with production of down-regulatory cytokine IL-10 occurred just prior to peak microfilaremia. Histological analysis revealed progressive inflammation of the lymphatic vessel walls, with intimal thickening and disorganization of collagen fibers. Inflammation was observed as early as 8 weeks post-infection and extended into the perivascular and subcutaneous tissues by 16 weeks post-infection. Finally, we developed a novel ferret PET/CT lymphoscintigraphy method demonstrating substantial changes in lymphatic anatomy and function as early as 3 weeks post-infection, with progression over the course of infection.

**Conclusions/Significance:**

*B*. *malayi* infection of ferrets is a robust model of human lymphatic filariasis that can be utilized to study efficacy of novel antifilarial agents against adult worms residing within lymphatic vessels. In conjunction with PET/CT lymphoscintigraphy, this model can also be used to investigate pathogenesis of lymphatic dysfunction in lymphatic filariasis and efficacy of medications aimed at reversing lymphatic dysfunction after clearance of adult worms.

## Author summary

Filariae are tissue-invasive parasitic roundworms transmitted by insects. In human infection, the species *Brugia* and *Wuchereria* cause leg swelling by disrupting the function of lymphatic vessels. These agents of lymphatic filariasis infect ~65 million people worldwide, causing genital and lower extremity (elephantiasis) swelling. Currently, there is a global program to eliminate lymphatic filariasis by treating all individuals living in at-risk countries with medicines against the parasites. However, these drugs do not kill adult worms when given as a short course and must be repeatedly administered once to twice a year for 5–10 years. Due to this limitation, there are ongoing efforts to develop new anthelmintics against the parasites causing lymphatic filariasis. Limitations with current small mammal models of lymphatic filariasis include difficulties in quantifying adult worm burdens and in assessing lymphatic vessel anatomy and function. In the 1980s it was shown that infection of ferrets with *Brugia* worms causes intralymphatic infection modeling human disease. Re-establishment of this model will enable us to test how well new anthelmintics work against the parasites in an intralymphatic infection model and will assist in determining whether new drugs alter lymphatic function. Here, we show that ferrets can be reproducibly infected with *B*. *malayi* larvae. We have characterized the stages of infection, and developed an *in vivo* technique allowing for visualization of changes in lymphatic anatomy and quantitative assessment of lymphatic function over the time course of infection.

## Introduction

The causative agents of lymphatic filariasis (LF) infect over 68 million people, causing genital disease in 19 million and lymphedema/elephantiasis in approximately 17 million [1]. An obstacle to current eradication efforts is the inability of antifilarial medications to effectively kill adult worms when given as a short course therapy. Consequently, there have been recent efforts to develop macrofilaricidal therapies that would be safe and effective when given for a short period of time [2–4]. Typically, the initial screening of drug candidates is performed *in vitro*, followed by *in vivo* testing in animal models. While there are several rodent models of filaria infection, current models in which adult worms reside in lymphatic vessels are limited by difficulties in quantifying adult worm burdens and in assessing lymphatic anatomy and function. Establishment of a model that overcomes these limitations would enable more robust efficacy testing of candidate drugs against filariae residing within lymphatic vessels as well as safety testing to ensure novel treatments do not worsen clinical disease.

In the 1980s, infection of ferrets with *Brugia* species was shown to cause a lymphatic infection which models human disease [5]. After injection of infectious stage L3 larvae into the footpad of a ferret, both *B*. *malayi* and *B*. *pahangi* develop to adult worms in the popliteal and inguinal lymphatics of the injected limb and release microfilariae from 3 to 8 months after infection [6]. Whereas single injections of 90–200 L3 larvae cause histologic lymphangitis, repeated injections of 25–30 L3s over several months cause clinically apparent lymphedema [7, 8]. To fill the need for a small mammal model of lymphatic filariasis, we have reestablished and further evaluated the *Brugia*/ferret model.

In this study, we show that ferrets can be reproducibly infected with *B*. *malayi* L3 larvae. We have identified the timecourse of microfilaremia, demonstrated changes in lymphatic vessel pathology, and confirmed that multiple infections can confer clinical lymphedema. Additionally, we have assessed the immunological changes that occur over time in the *Brugia*/ferret model. Finally, we have developed an *in vivo* technique of PET/CT lymphoscintigraphy in ferrets that allows for visualization of changes in lymphatic anatomy and quantitative assessments of lymphatic function over the time course of ferret infection.

## Materials and methods

### Ethics statement

All experiments were performed under an animal protocol approved by the Uniformed Services University’s Institutional Animal Care and Use Committee, which adheres to the United States Department of Agriculture’s Animal Welfare Regulations and Public Health Services Policy on Humane Care and Use of Laboratory Animals published by the National Research Council (8th Edition, 2011), as well as the Animal Research: Reporting of In Vivo Experiments (ARRIVE) guidelines.

### Ferret infection

Outbred ferrets were obtained from Marshall BioResources and maintained at the Uniformed Services University. Male ferrets were neutered prior to shipment. Animals were housed in groups of 2–3 males or females per cage in multilevel ferret cages, given free access to food and water, and allowed open playpen exercise at least once a week.

Infective L3-stage *Brugia malayi* larvae (L3s) were obtained from TRS Laboratories (Athens, GA). Ferrets 6 to 12 months of age were subcutaneously injected with 150 L3s in the right hind-footpad, and received a 200 microliter injection of phosphate buffered saline (PBS), pH 7.4, in the left hind-footpad as a vehicle control. Efficacy of infection was determined by the presence of circulating microfilaria in blood samples collected 11 to 14 weeks’ post-injection.

### Assessment of *B*. *malayi* patency

Beginning 10-weeks post *B*. *malayi* infection, 1 ml of blood was drawn from the cranial vena cava of anesthetized animals and transferred to EDTA-coated tubes. Blood samples were treated with ACK lysing buffer and passed through 3 micron Nuclepore Track-etch filter membranes (filters: Whatman #110612; filter holders: Whatman #420200) for microfilaria (Mf) collection. Filters were transferred to microscope slides and processed with the Richard-Allan 3-step stain protocol for fixation and staining (ThermoFisher Scientific #3300). Mfs per milliliter of blood were counted via light microscopy.

### White blood cell differential counts

One milliliter of blood was drawn from the cranial vena cava of anesthetized animals and transferred to EDTA-coated tubes to prevent clotting. Analysis of complete blood cell counts and white blood cell differential analyses was performed at VRL Animal Diagnostic (Gaithersburg, MD).

### Preparation of soluble worm antigen

*B*. *malayi* worms were suspended in phosphate-buffered saline (PBS), pH7.4 and homogenized using a MP FastPrep-24 Homogenizer (3 x 20 seconds at 4.0 meters/second) with 1.4 mm ceramic spheres (ThermoFisher Scientific # MP116913100). Homogenates were centrifuged at 12,000 *g* for 30 min at 4°C; supernatants were collected and filtered through 0.22-μm polyethersulfone (PES) membranes (EMD Millipore # SLGPM33RS). Concentration of the soluble protein content was determined using the BCA Protein Assay kit (ThermoFisher Scientific #23225). Samples were stored at -80°C prior to use.

### Measurement of parasite-specific immunoglobulin

At 2-week intervals, ferret blood samples were collected in heparinized plasma separator tubes (BD Microtainer #365985) and centrifuged at 15,000 *g* for 2 minutes. Plasma was collected and stored at -20°C until study end.

*B*. *malayi*-specific Immunoglobulin G levels were determined by sandwich enzyme-linked immunosorbent assay (ELISA). In brief, half-well plates (Corning #3688) were coated with 20 μg/ml of soluble *B*. *malayi* antigen (BmAg) for the capture of parasite-specific antibodies contained within ferret plasma samples. Following plasma incubation and several well washes, biotinylated goat anti-ferret IgG antibody (Rockland Immunochemicals Inc. #618-106-012), alkaline phosphatase-conjugated streptavidin (Jackson ImmunoResearch Laboratories) and 4-Nitrophenyl-phosphate disodium salt hexahydrate (4-NPP; Sigma #N4645-1G) were utilized for detection of the captured antibodies. Absorbance of the 4-NPP was measured at 405 nm (Victor^3^V; Perkin Elmer, Waltham, MA).

### PET and CT analysis of lymphatic vasculature

Ferrets were imaged pre- and post-infection as indicated in the text, using positron emission tomography (PET) and computerized tomography (CT). Scans were obtained using a custom-built ferret pallet compatible with the ASI Animal Handling System (ASI Instruments, Inc., Warren, MI) of the Siemens Inveon Multimodality scanner (Siemens Healthcare GmbH, Erlangen, Germany). In brief, anesthetized animals received a subcutaneous intra-digital injection (in the right hind-foot) of ^18^F-FDG (100–150 uCi), and PET data was acquired in list mode for 60 minutes. The frames were reconstructed for kinetic analysis, without scatter or attenuation correction, using a 3D-OSEM/MAP algorithm. Immediately following the PET scan, CT data was acquired. The PET and CT datasets were manually registered using fiducial markers and displayed as maximum intensity projection (MIP) images. Anatomical Regions-of-Interest (ROIs) were determined using the CT images, and the kinetics of ^18^F-FDG uptake through the lymphatic vasculature is reported as a percent of total injected tracer contained within the ROI over time.

### Adult *B*. *malayi* recovery counts

At study end points, anesthetized ferrets were euthanized via intracardiac injection of pentobarbital and phenytoin (Euthasol). Lymphatic vessels and surrounding soft tissues were dissected and removed from ankle-to-thigh of both the right (L3 infected) and left (PBS injected) hind-legs, and placed in RPMI 1640 media (Corning #15–040). Lymphatics were incubated at 37°C for ~2 hours, allowing worms to migrate from the vessels into the media. Adult worms were separated using fine tipped forceps, and counted with the aid of a dissecting microscope.

### Assessment of lymphatic inflammation

Following euthanasia of ferrets, lymphatic-laced dermal sections were isolated from the inner mid-calf of both the right (L3 infected) and left (PBS injected) legs. Sections were fixed in 10% formalin for 18 hours and then transferred to 70% ethanol. Tissue sections were mounted on microscope slides and stained with haematoxylin & eosin for assessment of inflamed lymphatic epithelium (including measurement of lymphatic wall thickness) and the presence of granulomas, plasma cells, eosinophils, and/or adult worms. Pathologists (J.J.F) and trainee pathologist (C.P.M.) performed blind assessments.

### Splenocyte and inguinal node lymphocyte isolation

Following euthanasia, the spleen and inguinal lymph nodes (right and left) were removed and individually placed in 5 ml of RPMI 1640 media (Corning #15–040). Tissue was dissociated by smashing with a 3 ml syringe plunger; cell suspensions were washed and pelleted. Red blood cells in the splenocyte suspension were lysed with ACK buffer. Splenocytes and lymphocytes were passed through a 70-micron nylon cell strainer (BD Falcon #352350) and resuspended in RPMI 1640 media supplemented with 10% fetal calf serum (Valley Biomedical #BS3032), 1% L-glutamine (Sigma #G7513), 10,000 IU/ml Penicillin, and 10 mg/mL Streptomycin (MP Biomedicals #1670249). Cells were counted and resuspended at appropriate concentrations. Cells not used immediately were stored in liquid nitrogen.

### Assessment of lymphocyte proliferation

Single-cell suspensions of splenic or lymph node cells were plated in duplicate at a concentration of 2×10^6^ cells/ml in a volume of 100 μl RPMI 1640 media (Corning #15–040) supplemented with 10% fetal calf serum (Valley Biomedical #BS3032), 1%l-glutamine (Sigma #G7513), 10,000 IU/ml Penicillin, and 10 mg/mL Streptomycin (MP Biomedicals #1670249). Cells were cultured at 37°C in 5% CO_2_, in the presence of 5 μg/ml Concanavalin A (ConA; Sigma #C5275), 20 μg/ml soluble *B*. *malayi* antigen, or without stimulation. After 40 hours, bromodeoxyuridine (BrdU) was added and cells were cultured for an additional 16 hours. Cell proliferation was assessed using a BrdU chemiluminescent assay per the manufacturer’s instructions (Roche Diagnostics #11647229001).

### Cytokine transcript analysis

Single-cell suspensions of splenic or lymph node cells were plated at a concentration of 1×10^6^ cells/ml in 1 ml RPMI1640 media (Corning #15–040) supplemented with 10% fetal calf serum (Valley Biomedical #BS3032), 1% l-glutamine (Sigma #G7513), 10,000 IU/ml Penicillin, and 10 mg/mL Streptomycin (MP Biomedicals #1670249). Cells were cultured at 37°C in 5% CO_2_, in the presence of 5 μg/ml Concanavalin A (ConA; Sigma #C5275), 20 μg/ml soluble *B*. *malayi* antigen, or without stimulation, and harvested after 72 hours. Total RNA was isolated from cells via Trizol lysis and RNeasy purification (Qiagen #74136). The mRNA contained in 25 ng or 250 ng total RNA (as noted in the text) was reverse transcribed using SuperScript IV (ThermoFischer #18091050) and the resulting cDNA pools were used as template for TAQman multiplex quantitative real-time PCR (qRT-PCR). Primer/ probe cocktails were specific for ferret cytokines IFNγ, IL-2, IL-4, and IL-10. Standard curves were generated by plotting known concentrations of cytokine-specific DNA plasmids against their calculated Ct values.

### Statistics

Analysis was performed with GraphPad Prism software (GraphPad Software, San Diego, Calif) to determine statistical significance. Differences between multiple groups were determined using the Kruskal-Wallis test, followed by Dunn post hoc multiple comparisons. Differences between two unpaired groups were determined using Mann-Whitney analysis. Differences between two paired groups were determined using Wilcoxon matched-pairs signed rank test. A *P* value of less than .05 was considered significant.

## Results

### *B*. *malayi* adult worms reside in the draining lymphatics of the inoculated limb

Male and female ferrets were infected by subcutaneous injection of 150 L3 stage *B*. *malayi* larvae into the right hind-leg footpad of each animal. At 8–16- and 28-weeks post-infection (PI) a subset of ferrets was euthanized and immediately analyzed for adult worm recovery.

**Fig 1:**
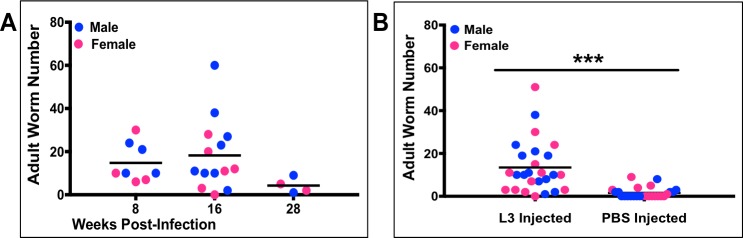
Adult *B*. *malayi* worm recovery from infected ferrets. A) Number of adult worms recovered from animals euthanized at 8 (n = 8), 16 (n = 14), and 28 (n = 4) weeks’ post-infection. Not statistically significant by Kruskal-Wallis test, followed by Dunn post hoc multiple comparisons. B) Number of adult worms recovered from the lymphatic vessels of the leg into which larvae were injected versus number of adult worms recovered from the PBS injected leg; n = 26 per group; ***p < 0.001 (Mann-Whitney test). Blue and pink circles indicate male and female ferrets, respectively.

Adult worms were recovered from ferret lymphatic vessels at all three time points indicating success of both development from larvae-to-adult stage as well as worm migration to (and residence in) the draining lymphatic system, as is typical of human lymphatic filariasis. A mean of 18 adult worms per ferret were recovered at 8 weeks PI and 20 worms per ferret at 16 weeks PI (Fig 1A). By 28 weeks, however, worm numbers decreased to a mean of 4 per animal. Significantly more adult worms were recovered from the femoral and popliteal lymphatics of the leg that was injected with L3 larvae (the right side); although some cross migration into the lymphatics draining the other hind limb was detected (Fig 1B). Extensive dissections were done on the first fifteen ferrets to determine if adult worms migrated to other body sites. Tissues evaluated included the inguinal lymphatics, abdominal cavity, thoracic cavity, and the heart. With the exception of one worm found in the inguinal lymphatics of one ferret, no worms were found outside the femoral and popliteal lymphatics. With regards to gender of host, no significant difference in adult worm recovery was observed between male and female ferrets.

### Microfilaremia of *Brugia malayi* infection coincides with eosinophilia and production of parasite-specific IgG

Blood samples were collected from each ferret prior to infection and at 2-week intervals following *B*. *malayi* L3 injections for analysis of white blood cell (WBC) differentials and production of parasite-specific IgG. Starting 10 weeks PI, each blood sample was analyzed for the presence of microfilariae (Mfs). Sampling continued through study end-points.

**Fig 2:**
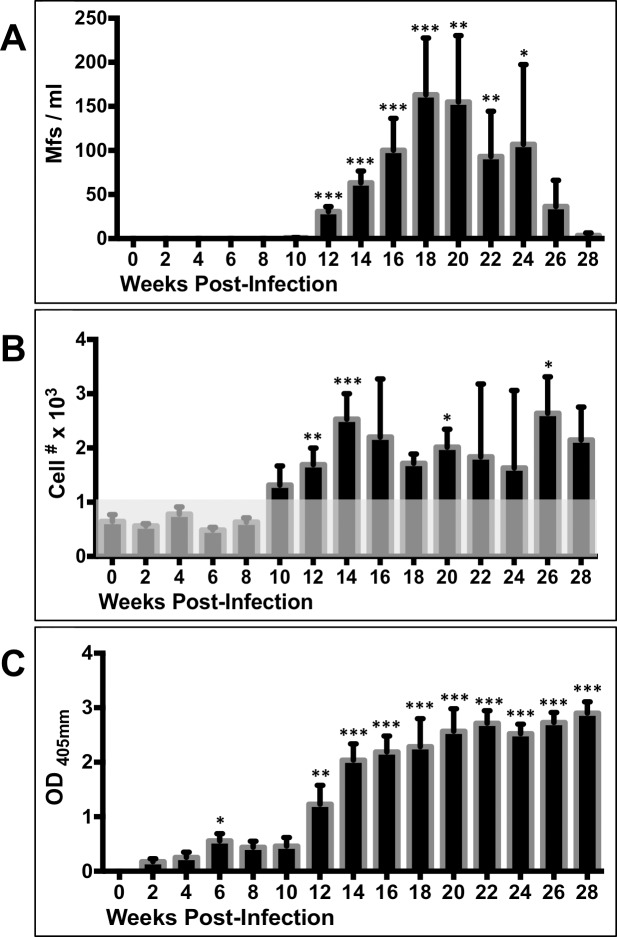
Timecourse of microfilaremia, eosinophilia and plasma levels of BmAg-specific IgG in *B*. *malayi*-infected ferrets. The mean and SEM values of (A) microfilaria per milliliter of blood, (B) eosinophil numbers per microliter of blood (shaded box indicates the normal cell range for ferrets), and (C) BmAg-specific IgG levels produced following *B*. *malayi* infection. Weeks 0 to 8 PI, n = 12; weeks 10 to 16 PI, n = 8; weeks 18–28, n = 4. Baseline values of Mfs/ml, eosinophil numbers, and antibody ODs were compared to corresponding values at the indicated post-infection timepoints for statistical significance; *p< 0.05, **p<0.01, ***p<0.001 (Kruskal-Wallis test, followed by Dunn post hoc multiple comparisons).

Patency onset was observed by 12 weeks PI, in both male and female ferrets; with peak microfilaremia occurring between 16- to 20-weeks PI (Fig 2A). Substantial variation of microfilaria numbers was noted between animals; with peak counts in male ferrets ranging from 11–378 Mfs/ml of blood and peak counts in female ferrets from 15–615 Mfs/ml. To address potential periodicity of Mf concentrations, we compared microfilaria counts from morning (9 a.m.) versus night (11 p.m.) blood draws in a subset of animals, but observed no significant differences (S1 Table). There were no differences in the adult worm burdens of male and female ferrets euthanized at 16 weeks (mean # adult worms: 18 per male ferret and 19 per female ferret).

The onset of eosinophilia, defined in ferrets as >1.1x10^3^ cells /microliter blood, correlated with that of patency at 12 weeks PI. Eosinophil levels remained elevated until study endpoint (Fig 2B). A rapid increase of *B*. *malayi* antigen-specific IgG was also detected in the plasma beginning at 12 weeks PI (Fig 2C).

### Analysis of ex-vivo cellular response to *B*. *malayi* antigen stimulation

**Fig 3:**
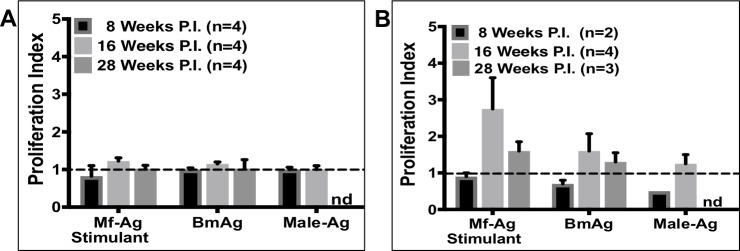
*Ex vivo* stimulation of lymphocytes with *B*. *malayi* antigen induces proliferation. Proliferation of (A) Splenocytes and (B) draining lymph node cells in response to stimulation with microfilariae antigen (Mf-Ag), gravid female antigen (BmAg), or adult male antigen (Male-Ag). Shown here are the mean and SEM values. nd = no data. Not statistically significant by Kruskal-Wallis test, by Dunn’s multiple comparisons (Mf-Ag and BmAg comparisons), nor by Mann-Whitney (Male-Ag comparisons).

To assess splenocyte and draining lymph node cell proliferation in response to filarial antigen stimulation, cells were cultured for 72 hours in the presence of 20 μg/ml soluble *B*. *malayi* antigen and analyzed for the incorporation of BrdU using a chemiluminescent assay. Antigen was prepared from *B*. *malayi* microfilariae (Mf-Ag), adult female worms containing *in utero* microfilariae (BmAg), or adult male worms (Male-Ag). The proliferation index was calculated as the optical density of antigen stimulated cells divided by the optical density of control cells (media exposure alone). Splenocytes removed from animals at 8, 16 or 28 weeks PI displayed no substantial proliferation in response to any of the parasite antigens (Fig 3A). In contrast, cells collected from the draining popliteal lymph nodes exhibited substantial proliferation in response to MF-Ag and BmAg, especially at 16-weeks PI (Fig 3B). At this timepoint, proliferation of stimulated cells was significantly more robust than cells cultured in media alone (media vs. Mf-Ag p = 0.002, BmAg p = 0.021, and Male-Ag p = 0.002). These results suggest that localized immune responses are greater than systemic ones in lymphatic filariasis.

To examine potential differences in cytokine production in response to parasite antigen at varying stages of infection, splenocyte transcript levels were analyzed using Taqman qRT-PCR. RNA was isolated from cultured cells following 72 hours of incubation in the presence of *B*. *malayi* antigen (20μg/ml BmAg). cDNA pools were generated and used as template for detection of inflammatory (IFNγ, IL-2, IL-4) and anti-inflammatory (IL-10) cytokines. Standard curves were generated from known concentrations of cytokine-specific DNA plasmids, thus allowing for the calculation of picograms of mRNA per cell.

**Fig 4:**
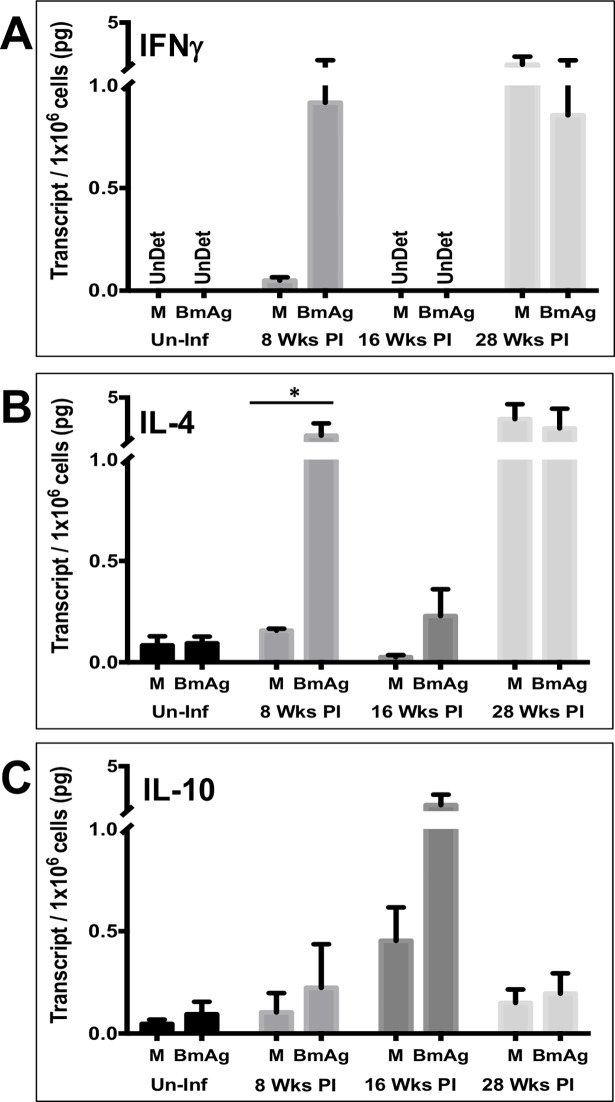
*Ex vivo* stimulation of splenocytes with BmAg reveals that modulation of cytokine gene transcription is dependent on duration of *B*. *malayi* infection. Cytokine transcription profiles, IFNγ (A), IL-4 (B), and IL-10 (C) of splenocytes stimulated for 72 hours with or without 20 μg/ml of BmAg (antigen derived from gravid female worms). M = Media alone; ND = Not Detected; n = 4 for each data set. Differences in transcript amounts produced by stimulated versus unstimulated lymphocytes were analyzed for statistical significance; *p< 0.05 (Mann-Whitney test).

At 8-weeks post-infection ferrets displayed a mixed Th1/Th2 cytokine profile, with increased transcription of both IFNγ and IL-4. By 16 weeks, there is evidence of marked immunoregulation with increased transcription of the down-regulatory cytokine IL-10 and decreased transcription of IFNγ and IL-4 (Fig 4A–4C). At 28 weeks PI, a timepoint by which almost all animals had cleared their microfilaremia, IL-4 and IFNγ transcription is elevated again and IL-10 is markedly decreased. The high transcript level of both IL-4 and IFNγ observed even in cells that were not given ex-vivo stimulation with BmAg suggest high production of both of these cytokines *in vivo* at the 28 week timepoint. It is interesting to note that the time of peak immunoregulation (16 weeks) occurs just prior to the time of peak microfilaremia, and that timing of Mf clearance at 28 weeks is associated with resolution of immunoregulation.

### Histological analysis of lymphatic vasculature

**Fig 5:**
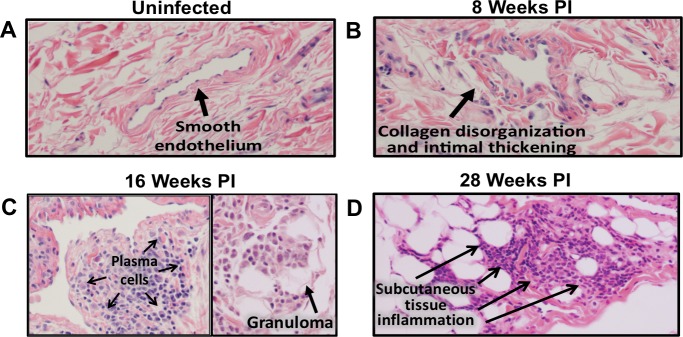
Histology of lymphatic vessels and surrounding tissue. (A) un-infected controls (20x), (B) ferrets infected for 8-weeks (40x), (C) 16-weeks (20x), and (D) 28-weeks (20x). Formalin treated tissue samples were stained with hematoxylin and eosin.

**Fig 6:**
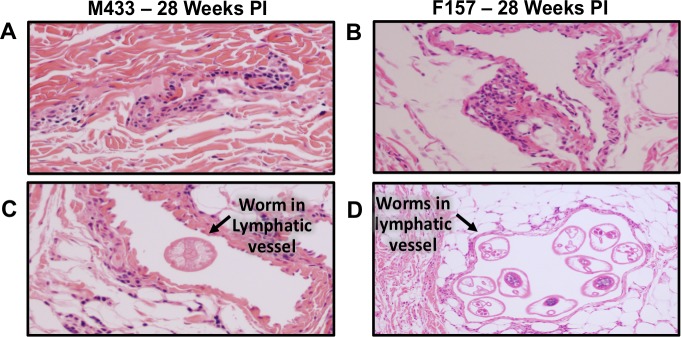
Histology of lymphatic vessels and surrounding tissue from lymphatic vessels without (A, B - 20x) and with (C -20x, D - 10x) resident adult worms. Formalin treated tissue samples were stained with hematoxylin and eosin.

Post-mortem tissue samples were collected for histological analysis of the lymphatic vasculature. Fig 5A shows the endothelium of a medium sized lymphatic vessel from a healthy, uninfected ferret. In contrast, the lymphatic vessels from an animal 8 weeks PI show disorganization of the collagen fibers and intimal thickening (Fig 5B). This is more pronounced in the 16-week infected animal (Fig 5C), with increased inflammation effacing the normal vessel wall architecture. Although the inflammation is most striking in the lymphatic walls, perivascular and subcutaneous inflammation is present and comprised primarily of plasma cells and histiocytes with eosinophils and neutrophils present in smaller quantities. Although the inflammation extending outside the lymphatic vessel into the surrounding tissue is evident at 16 weeks PI (Fig 5C—right panel) it is more prominent in the 28 weeks infected animal (Fig 5D). Interestingly while there is inflammation observed in the wall of multiple lymphatic vessels, it appears less evident in the walls of vessels containing adult *B*. *malayi* (Fig 6A–6D). Despite the decreased inflammation in vessels immediately surrounding worms, pathologic changes of collagen thickening and disorganization remain discernible.

Measurements of the lymphatic wall diameter were taken for a subset of infected animals. Comparisons were made between samples from the L3 versus PBS injected sides, as well as between stages of infection (S1 Fig). The most severe change in vasculature thickness was observed at 16 weeks PI, with a significant difference noted between vessels of the right and left legs.

### Assessment of lymphatic anatomy and function by ^18^F-FDG PET/CT imaging

**Fig 7:**
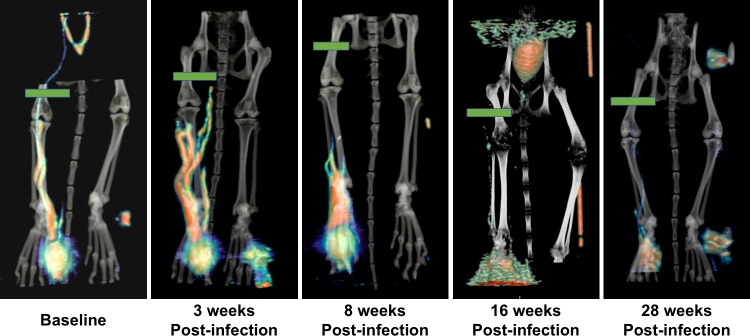
Visualization of lymphatic anatomy using PET imaging. PET and CT images were acquired from ferret F814 pre-infection and 3-, 8-, 20, and 28-weeks post *B*. *malayi* infection. The portrayed maximum intensity projection (MIP) images were constructed by collapsing data from 60 individual PET files (each consisting of a one-minute scan) into a single static frame. The resulting PET image was aligned to the corresponding CT image. Green bars show the designated Region-of-Interest used for kinetic analysis of tracer flow depicted in Fig 8.

To assess the effects of filarial infection on lymphatic anatomy and function, we developed a technique to perform ferret PET/CT lymphoscintigraphy. Following subcutaneous injection of ^18^F-FDG (fluorodeoxyglucose) into the right hind foot of the ferret, consecutive PET images were captured for 60 minutes using a Siemens Inveon Multimodality scanner. A series of ^18^F-FDG PET/CT lymphoscintigraphy images from ferret F814 is shown in Fig 7 and demonstrates changes in the lymphatic anatomy at various timepoints over the course of a 28-week infection. The baseline image, obtained prior to infection, clearly shows narrow lymphatic channels running up the lower leg. Three weeks after infection, the lymphatic channels appear slightly dilated and diverted tracer can be seen flowing through collateral channels (adjacent to the tibia and fibula), indicative of obstruction within the main lymphatic vessel(s). By 8-weeks post-infection there is discernable pooling of tracer near the ankle, demonstrating further decline in flow. Images taken at 20 and 28 weeks PI show dramatic loss of lymphatic function with little to no tracer uptake from the foot. This animal was amicrofilaremic by 26 weeks PI. This technique is substantially more sensitive for assessment of lymphatic function than clinical inspection as this animal was asymptomatic with regard to measurable lymphedema throughout the study.

**Fig 8:**
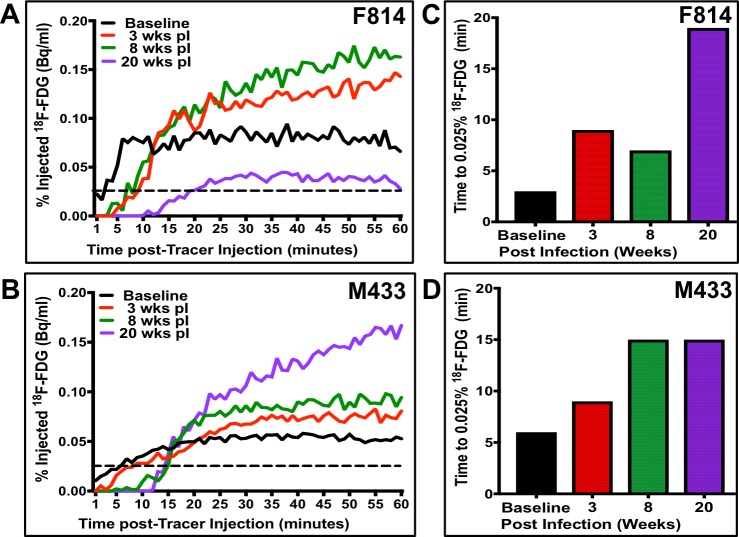
Kinetic analysis of PET imaging demonstrates lymphatic dysfunction at all timepoints post-infection. Detection over time of tracer-containing lymph at the mid-femur ROI for ferrets F814 (A) and M433 (B)—signal is reported as % of total injected ^18^F-FDG. (C-D) Elapsed time for tracer to reach 0.025% of the total injected at the ROI. Dashed line indicates 0.025% threshold used for elapsed time analysis.

A key feature of lymphatic health is the steady flow of lymph and, thus, the degree of vessel dysfunction can be inferred by delays of tracer uptake to a proximal region. We quantified the degree of lymphatic dysfunction by plotting the kinetics of accumulated tracer intensity over time at the mid-femur, which we established as our designated region-of-interest (ROI, green bars in Fig 7). We chose this location as it overlaps the lymphatic vessels where most adult worms reside and because it can be reproducibly identified by CT imaging at each analysis timepoint. Fig 8A and 8B show the graphed kinetics from ferrets F814 (a microfilaremic animal from weeks 12 to 24 PI) and M433 (microfilaremic from weeks 10 through 28 PI). Function was primarily determined by assessing the time it takes for ^18^F-FDG to reach the mid-femur ROI following injection of the tracer into the foot. For both animals, there is evident delay in uptake as early as 3 weeks PI compared to baseline. Using 0.025% of the total ^18^F-FDG signal as a ROI detection threshold, prior to *B*. *malayi* infection it took the tracer 2 minutes in ferret F814 and 6 minutes in ferret M433 to reach the mid-femur. In contrast, at 3 weeks PI it took 9 minutes after tracer injection for 0.025% of the signal to be detected at the ROI of either ferret, and 15–20 minutes post-injection at 20 weeks PI (Fig 8C and 8D). These findings demonstrate that subclinical lymphatic dysfunction occurs very rapidly in the setting of infection with lymphatic filariae, and that dysfunction continues to decline over the first few months.

In addition to time of uptake, we also assessed peak tracer intensity at the mid-femur. At analyses time points 3- and 8-weeks PI for F814, and 3-,8-, and 20-weeks PI for M433 the tracer intensities reach levels higher than baseline (Fig 8A and 8B). This suggests that there is lymphatic dysfunction occurring proximal to the ROI causing an impediment of drainage from the mid-femur region. At 20-weeks PI, tracer in ferret F814 does not reach baseline intensity during the scan duration, suggesting severe lymphatic dysfunction that is markedly limiting flow of lymphatic fluid to the ROI.

## Discussion

In this study, we have re-established *Brugia malayi* infection of ferrets as a model of lymphatic filariasis and demonstrate that it has parasitological, immunological, and histological parallels with human infection. Our findings are consistent with those of the original studies in regard to patency onset, transient microfilaremia, the timing of eosinophilia, and the average number of adult worms recovered [6]. Further, we have developed a novel PET/CT lymphoscintigraphy technique in ferrets that enables monitoring of lymphatic anatomy and function in the same animal over time. This technique demonstrated that even when lymphedema is not clinically apparent, lymphatic filariasis causes dramatic changes in lymphatic anatomy and function within weeks of infection.

One of the advantages of this model is that it results in patent (microfilaremia-causing) infection in which adult worms reside predominantly within the draining lymphatics of the inoculated limb. In this study, ~90% of adult worms were found in the inguinal and femoral lymphatics draining the infected limb and the remainder found in the draining lymphatic vessels of the contralateral side. All animals developed microfilaremia, with patency lasting from week 12 to ~26 weeks post-infection.

These results are in contrast to immunocompetent mice, which do not develop microfilaremia after infection with either *Brugia malayi* or *Brugia pahangi* [9, 10]. *B*. *malayi* does cause patent infections in jirds (Mongolian gerbil, *Meriones unguiculatus*) [11, 12] and *Mastomys* [13, 14], but the adult worms end up residing in the heart, lungs, and peritoneal cavity as well as lymphatic vessels. This makes enumeration of adult worm numbers challenging for studying efficacy in vaccine or treatment studies. Additionally, while both animals exhibit histological changes, neither develops clinical disease.

As is observed in human LF, *B*. *malayi* infected ferrets exhibit an initial mixed Th1/Th2 immune response, as evidenced by increased cytokine transcription (IL-4, IFNγ) and peripheral eosinophilia, followed by later evidence of immunoregulation. The immunoregulatory response appeared to peak at 16 weeks PI, with high levels of IL-10 in response to parasite antigen and a simultaneous decrease in the release of inflammatory cytokines from splenocytes after *in vitro* stimulation with parasite antigen. Interestingly, this timepoint immediately precedes maximal circulating microfilaria levels, suggesting that immunoregulation allows for microfilaremia. Indeed, we observed that when microfilaremia resolves, at 28-weeks post-infection, immune regulation is at its lowest. These results parallel what is observed in humans where individuals that are symptomatic typically have low levels of microfilaremia and high inflammatory cytokine responses to antigen whereas those that are asymptomatic microfilaremics typically are highly immunoregulated [15–21]. Because some antifilarial medications work with the immune system to enable parasite clearance [16, 22, 23], and because potential adverse effects of novel agents include inflammatory responses against dying adult worms, understanding whether the immune responses in the *Brugia*/ferret model parallel those in humans is important for determining whether this model will be a robust one for predicting effects of antifilarial medications in humans.

Popliteal lymph node cells collected from the infected leg 16 weeks PI proliferated in response to *B*. *malayi* antigen. Interestingly, the most robust response came with exposure to microfilarial antigens, closely followed by exposure to antigens from gravid (Mf containing) female worms. There was no response to adult male antigen. These results suggest that Mfs may be the primary inducers of a local immune response.

Our histological analysis of excised lymphatic vessels and the surrounding tissue revealed a number of changes similar to those seen in human studies, including cellular infiltrate, wall thickening, and vessel dilation. By 8 weeks’ post-infection lymphatic vessels of infected animals demonstrated disorganization of collagen fibers and intimal thickening. These changes became more severe by 16 weeks’ post-infection. Cellular infiltration of the vessel walls was composed primarily of histiocytes and plasma cells with eosinophils and neutrophils present in lower concentrations. Interestingly, we frequently observed cellular inflammation beyond the vessel walls. Currently, the prevailing view with regards to the development of clinical elephantiasis in patients with LF is that recurrent skin and soft tissue infections drive the tissue remodeling that lead to this clinical phenotype. Our observation that extra-luminal soft tissue inflammation frequently occurs in the absence of deep bacterial infections suggests that inflammation against the worms themselves may contribute to development of chronic soft tissue changes in LF.

To assess effects on the lymphatic system, we developed a PET/CT lymphoscintigraphy technique for studying lymphatic anatomy and function in ferrets. Reconstruction of 60 continuous minutes of PET imaging after subcutaneous injection of ^18^F-FDG provided highly detailed images of lymphatic vessel anatomy that enabled visualization of lymphatic vessel width and development of collateral vessels. Lymphatic function could be quantified by measuring tracer uptake over time at the mid-femur region after initial injection of tracer into the footpad. One of the most striking observations was the rapidity with which lymphatic dysfunction occurs. As early as 3 weeks post-infection we noted dilation of lymphatic vessels, a reduction in ascending lymphatic flow, and development of collateral vessels. This process appears to worsen throughout 20 weeks of infection. Interestingly, even though we observed marked reductions in tracer uptake at all timepoints following a single inoculation of L3 larvae, these animals did not display clinical disease. This is consistent with our understanding of human lymphatic filariasis. Filarial infection often occurs in early childhood, but manifestation of clinical lymphedema typically occurs later in life. However, detection of lymphatic obstruction in asymptomatic children has been documented using lymphoscintigraphy [24, 25]. Our PET/CT imaging data supports these findings, showing that changes in lymphatic function occur very early in the course of infection. Further, a recent study by S.K. Kar, *et al*. demonstrates via patient lymphoscintigraphy that annual treatments of DEC and albendazole during the early stages of LF infection, may reverse lymphatic pathology [26]. We envision lymphoscintigraphy analysis in the ferret/*Brugia* model as a valuable technique toward better understanding the mechanisms of LF-induced lymphedema, and for analysis of drug efficacy in the treatment of this disease.

Finally, consistent with prior studies done on ferrets [5, 8], we demonstrated that clinically apparent lymphedema disease can be obtained after multiple injections of infectious *B*. *malayi* L3 larvae (see S1 Text). In endemic regions, high exposure of carrier mosquitos is associated with increased rates of lymphedema [27]. Thus, multiple injections of L3 stage worms are a better mimic of natural infection conditions. In the ferret model, we were able to induce chronic measurable lymphedema in one of four animals, and transient pathology in one additional animal, by administering 6 subcutaneous injections of 30 L3 larvae each over a course of 12 weeks. Swelling of the ankle and knee regions was detected 20 weeks post-infection in 2 of 4 animals and persisted through the study endpoint at 52 weeks PI in one ferret. In the original study of repeated *B*. *malayi* infection in ferrets, pathology was noted in 14 of 18 animals followed for 1–2 years post-infection [8]. We suspect the low incidence of clinical lymphedema observed in our study may have been due to the shorter time period of post-infection analysis and the low numbers of animals studied.

We wish to acknowledge the following limitations to this study; foremost the low number of ferrets at the 28 week timepoint and in the multiple injection analysis (for both, n = 4). Additionally, limited immunological reagents are available for this animal model. Thus, we used RNA transcript levels to quantify cytokine production rather than directly measuring protein, as few ferret-specific antibodies are commercially obtainable. Further, ferrets are expensive to purchase and maintain. The animals used in this study were outbred and thus results were more variable than would be expected in inbred rodent studies.

We believe this model will be extremely useful to researchers developing new tools to treat and prevent lymphatic filariasis. Current single dose LF chemotherapeutic regimens used in the Global Program to Eliminate Lymphatic Filariasis, while effective for short-term elimination of microfilariae, are insufficient in killing adult worms. Thus, eradication efforts require repeated treatments of populations in endemic countries for many years. The *Brugia*/ferret model could be ideal for testing promising macrofilaricidal candidates as it would enable efficacy analysis of promising candidate drugs against a human agent of lymphatic filariasis residing within lymphatic vessels. In addition to evaluating efficacy, this model can also be used to ensure that new antifilarial agents do not worsen lymphatic function. Finally, this model can be useful for studying the mechanisms by which lymphatic dysfunction occurs in LF infection, and for investigating modalities aimed at improving lymphatic function once infection has been cleared.

## Supporting information

S1 Table Analysis of microfilaremic periodicity in ferrets. (TIF)

S1 Fig Thickening of lymphatic vasculature is altered with chronic *B*. *malayi* infection. (TIF)

S1 Text Clinical disease develops in ferrets repeatedly infected with *B*. *malayi* L3 larvae. (PDF)
