# Supplementary figures and images for: Brugia malayi infection in ferrets – A small mammal model of lymphatic filariasis
Source: PLoS Negl Trop Dis. 2018 Mar 30;12(3):e0006334. doi: 10.1371/journal.pntd.0006334 (PMC5895066; doi:10.1371/journal.pntd.0006334)

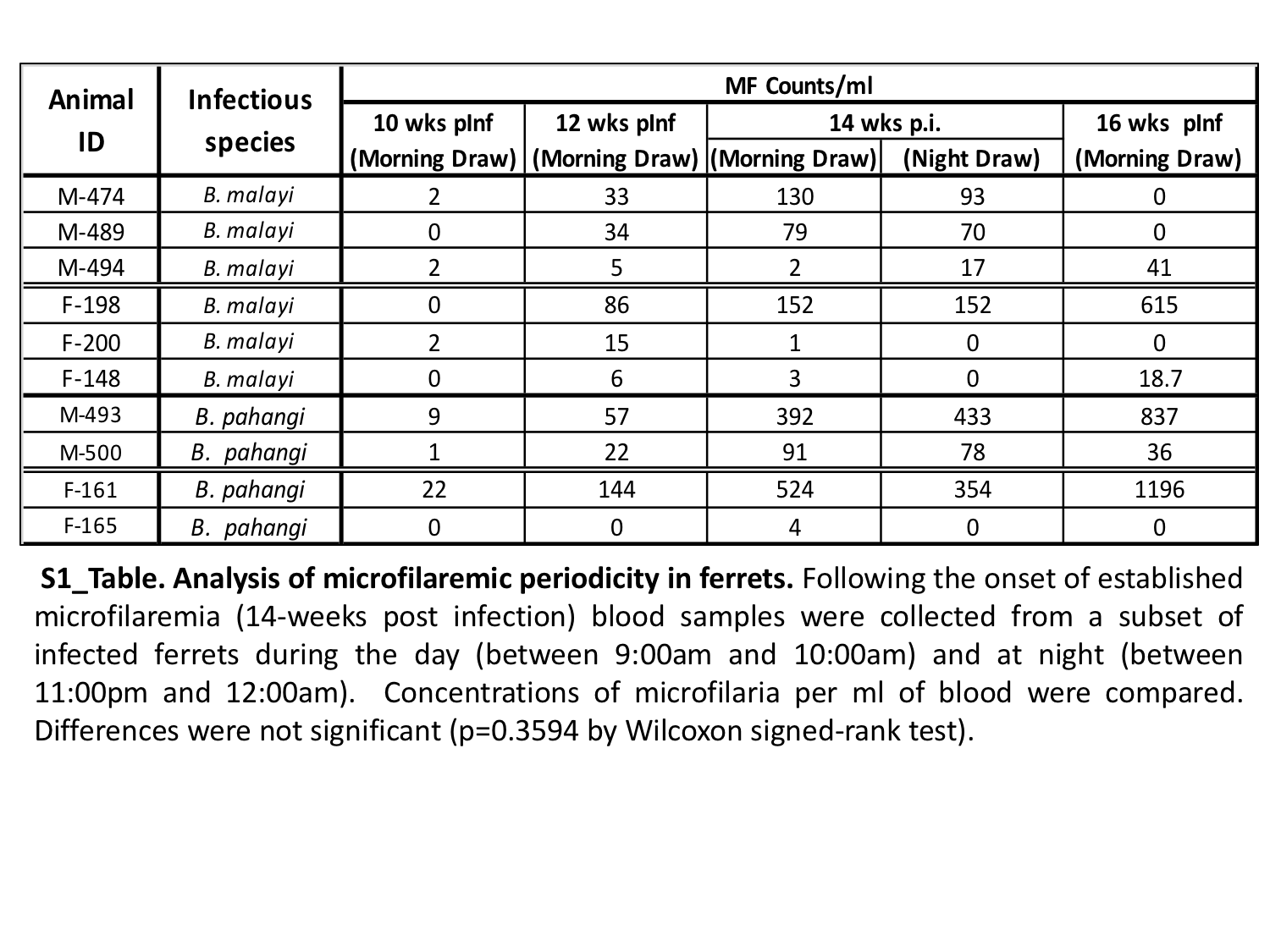

Supplement: S1 Table — (TIF) [file pntd.0006334.s001.tif]

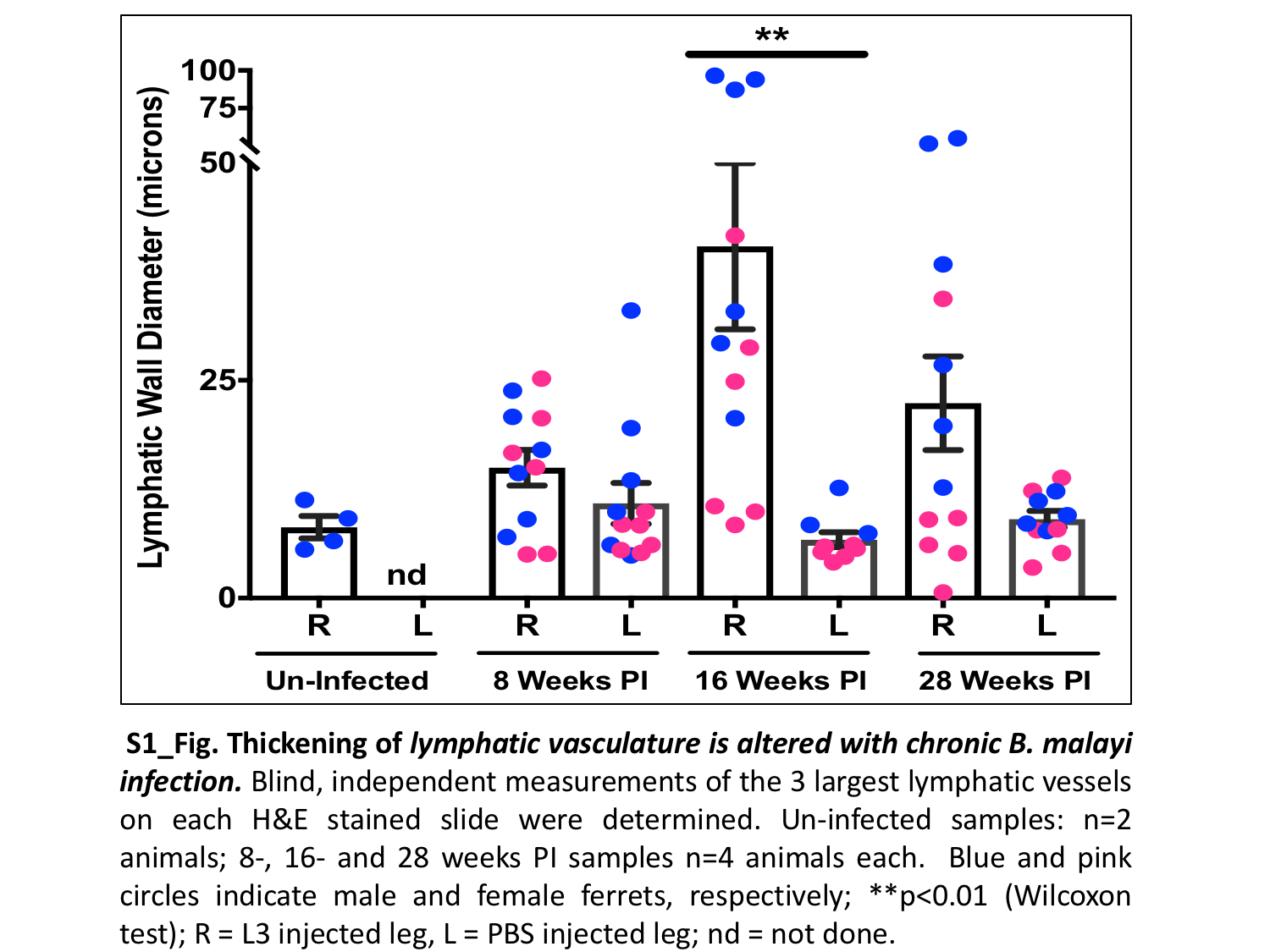

Supplement: S1 Fig — (TIF) [file pntd.0006334.s002.tif]
