# Supplementary material for: Brugia malayi infection in ferrets – A small mammal model of lymphatic filariasis
Source: PLoS Negl Trop Dis. 2018 Mar 30;12(3):e0006334. doi: 10.1371/journal.pntd.0006334 (PMC5895066; doi:10.1371/journal.pntd.0006334)
Supplement: S1 Text — (PDF) [file pntd.0006334.s003.pdf]

S1 File. Clinical Disease Develops in Ferrets Repeatedly Infected with *B. malayi* L3 larvae

Although we observed profound changes in lymphatic anatomy and function by both histology and PET/CT imaging, clinical lymphedema was not detected in any of the animals administered a single injection of *B. malayi* L3s. Because previous studies in the ferret model have shown that multiple injections can lead to measurable pathology [7], we evaluated for development of clinical disease in four ferrets repeatedly challenged by subcutaneous injection of 25 *B. malayi* larvae into the right hind paw on weeks 0, 2, 4, 6, 8, and 10. By 20 weeks following the initial infection, one of these ferrets developed obvious lymphedema in the injected limb (Fig A), a second animal developed mild but transient lymphedema, while the other animals remained asymptomatic (data not shown). In the earlier studies, over 10 challenges of L3 larvae were required for the induction of gross lymphatic pathology. Therefore, we resumed biweekly injections of *B. malayi* L3s for the 3 asymptomatic ferrets with administration at weeks 30, 32, 34, 36, 38, and 40. We followed all the animals receiving multiple injections through 50 weeks post the initial infection, and observed no additional lymphatic pathology.

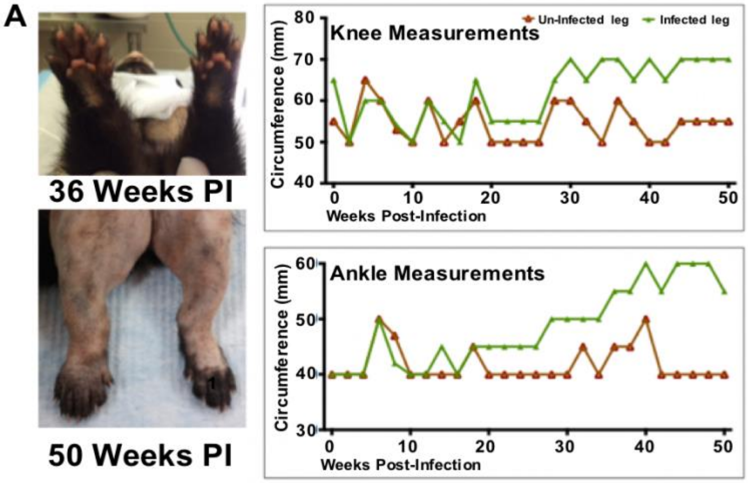

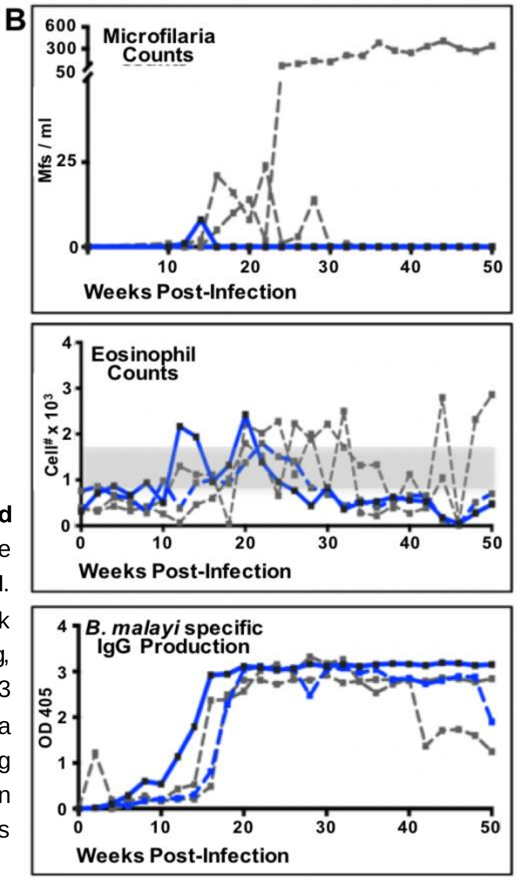

Clinical pathology can be induced in ferrets via repeated *B. malayi* L3 injections. (A) Circumference measurements at ankle and knee of ferret M498 reveal onset of lymphedema 20-weeks PI. Infection consisted of 6 injections of 25 L3 larvae given at 2-week intervals. Red and green lines represent the PBS or L3 injected leg, respectively. (B) Shown for each of 4 ferrets receiving multiple L3 injections are microfilaria/ml blood, eosinophils/∝l blood (shaded area indicates normal ferret range), and BmAg-specific IgG levels following infection. Upon necropsy, no viable adult worms were observed in these animals. The solid and dashed blue lines represent the animals with consistent (M498) and transient lymphedema, respectively.

As with ferrets given a single injection of L3s, microfilaremia (with one exception), eosinophilia and high levels of circulating BmAg-specific IgG developed 12 to 14-weeks PI (Fig B). It is interesting that mild or no microfilaremia was observed in the animals that displayed consistent and transient pathology, respectively. Of the ferret that maintained clinical disease, onset of lymphedema (20-weeks PI) occurred soon after the resolution of microfilaremia (16-weeks PI).
